# Nonlinear dimensionality reduction and Bayesian optimization for accelerating design of materials

**DOI:** 10.1038/s41598-026-51517-8

**Published:** 2026-05-09

**Authors:** Muhammad Osman Nadeem Farooqui, Isaac Y. Miranda-Valdez, Tero Mäkinen, Juha Koivisto, Mikko J. Alava

**Affiliations:** Department of Applied Physics, Aalto University, P.O. Box 15600, 00076 Espoo, Finland

**Keywords:** Dimensionality reduction (DR), Principal component analysis (PCA), T-distributed stochastic neighbor embedding (t-SNE), Uniform manifold approximation and projection (UMAP), Gaussian process (GP), Bayesian optimization (BO), Engineering, Materials science, Mathematics and computing

## Abstract

Optimizing biobased foam formulations is challenging because experiments are costly and fast-to-measure surrogate properties occupy high-dimensional spaces. Bayesian optimization (BO) with Gaussian process regression (GPR) can guide data-efficient searches, but its performance depends on the dimensionality of the inputs. Here, we evaluate nonlinear dimensionality reduction (DR) methods, namely t-distributed stochastic neighbor embedding (t-SNE) and uniform manifold approximation and projection (UMAP), in comparison with principal component analysis (PCA) for biobased foam optimization. Using an existing dataset comprising 26 distinct methylcellulose-cellulose fiber foam formulations with rheological and mechanical measurements, we first train a Gaussian process (GP) on low-dimensional representations of rheological observables. BO is then applied in a single-step, non-sequential manner on this fixed dataset, to evaluate the quality of the latent representations and identify high-yield-stress regions. A second GP maps foam-formulation compositions to the reduced rheological coordinates, enabling reconstruction of candidate formulations in the composition space. Across all DR methods, BO identifies similar high-performing formulations achieving yield stress values comparable to the experimentally validated optimum. PCA acts as a baseline due to its deterministic and hyperparameter-free nature, while nonlinear methods can achieve comparable performance when appropriately tuned. These findings demonstrate that nonlinear DR-assisted BO provides a data-efficient framework for optimizing rheology-governed soft-matter materials.

## Introduction

Designing biobased foams^1–11^ is constrained by long experimental feedback loops: while rheological characterization of the precursor suspension can be performed rapidly, evaluating the mechanical performance requires full sample manufacture and drying for each formulation. As a result, exploring composition-process-structure-property relationships is inherently data-limited. The resulting datasets are high-dimensional, often feature strong correlations^11^, and exhibit potentially nonlinear dependencies^12^ that complicate direct modeling. In this setting, black-box optimization methods that make efficient use of limited data are particularly valuable.

Bayesian optimization (BO)^13,14^, typically implemented using Gaussian process regression (GPR)^15,16^, and an acquisition function to guide active learning^17,18^, offers a principled approach for identifying high-performing formulations with few experimental evaluations. BO has been successfully applied across materials science, from computational alloy discovery^19–21^, and material parameter identification^22,23^ to broader process-structure-property optimization tasks^24–30^. Nevertheless, the performance of BO degrades in high-dimensional spaces^31,32^ due to difficulties in fitting surrogate models and optimizing nonconvex acquisition landscapes^33^. Dimensionality reduction (DR) offers a practical solution by transforming high-dimensional inputs into a lower-dimensional latent space. The simplest DR method, principal component analysis (PCA)^34,35^, provides a stable linear embedding and was recently^11^ combined with BO to accelerate the optimization of foams composed of methylcellulose (MC) and cellulose fibers. A wide variety of related linear DR techniques^36^, such as linear discriminant analysis^37^, independent component analysis^38^, and truncated singular value decomposition^39^, can also be used, but they share the same limitations as PCA. These methods work well when the data lies in or near a linear subspace, but they cannot capture nonlinear structure^36^. Nonlinear DR techniques, such as t-distributed stochastic neighbor embedding (t-SNE)^40^ and uniform manifold approximation and projection (UMAP)^41^, can uncover nonlinear manifold structure that PCA may miss. However, these methods do not provide native inverse mappings and may distort the global geometry^42–44^. Their compatibility with regression-based BO pipelines in small experimental datasets remains largely unexplored.

Here, we evaluate whether nonlinear DR methods can reliably support surrogate modeling and BO in an experimental soft-matter system. We use an existing experimental dataset from our previous study^11^, consisting of 26 unique methylcellulose-cellulose fiber formulations, each replicated five times to account for experimental variability, resulting in a total of 130 samples. Each formulation is characterized by 11 rheological observables (detailed in Supplementary Section S1) measured on the precursor suspensions, together with the yield stress of the corresponding dried foam samples. Rheological systems in general exhibit nonlinear behavior; however, the extent to which nonlinear structure manifests within a given experimental dataset is not known a priori and depends on the sampled composition and processing domain. In the present system, linear correlations dominate many rheological observables, while localized nonlinear relationships are also observed (Supplementary Section S1). This motivates a comparative evaluation of linear and nonlinear dimensionality reduction methods to assess whether nonlinear embeddings, when properly tuned, provide improved results compared to linear baseline PCA.

This work follows the same latent-space Bayesian optimization strategy introduced by Miranda et al.^11^. In addition to linear PCA embedding, we incorporate nonlinear dimensionality reduction techniques (t-SNE and UMAP) to assess whether nonlinear latent representations can reliably support regression and optimization in small experimental datasets. We compare these DR methods within a unified DR-GPR-BO framework. The first Gaussian process (GP) model is used to learn the relationship between latent rheological observables and yield stress, while a second GP model maps compositions into the latent space. This auxiliary model is interpreted as a smooth interpolator that enables reconstruction of candidate formulations identified via BO, rather than as a true inverse model. In the present study, BO is applied in a diagnostic manner on a fixed dataset, rather than within a closed-loop experimental framework. This design allows us to isolate and compare the impact of different DR methods without confounding effects from iterative data acquisition.

We assess embedding quality, surrogate predictive accuracy, optimization behavior, and agreement between reconstructed optima and experimentally validated compositions. Across all DR methods, we show that nonlinear embeddings can produce latent spaces suitable for regression and optimization when appropriately tuned, and that all approaches recover the same high-performing formulation previously identified using PCA-based BO^11^. These results indicate that the rheological feature space exhibits a strong low-dimensional structure and that DR-assisted BO is robust to the choice of embedding.

## Results

The overall goal of the optimization here is to obtain cellulose foams with a high mechanical strength. The foam manufacturing starts from liquid suspensions consisting of water, methylcellulose, and cellulose fibers. The rheological properties of the liquid suspension are characterized by rheometry, and various rheological observables (Supplementary Section S1) are determined from these experiments. Then the suspension is aerated to form a liquid foam and dried to form the final dry foam. The mechanical properties of the foam (e.g., the goal here, high yield stress) are characterized by performing compression tests on the foam samples. The manufacturing process and experimental work are described in detail in our previous works^5,11^.

**Fig. 1:**
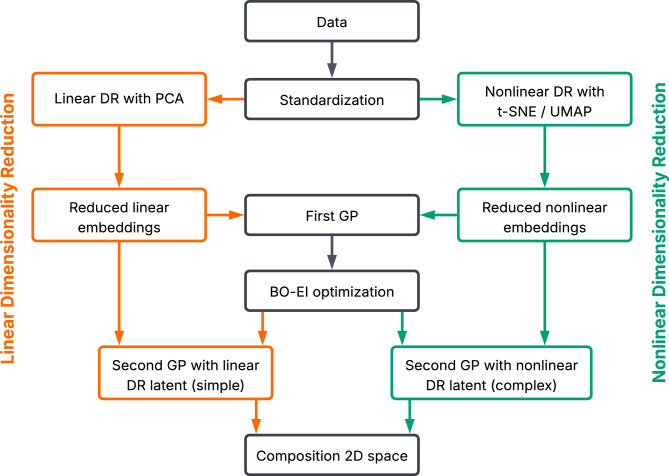
Overview of the DR-GPR-BO workflow deployed in this study. Standardized rheological features are embedded into low-dimensional latent spaces using linear (PCA, left panel) and nonlinear (t-SNE, UMAP, right panel) DR methods. In each latent space, a first GP model predicts mechanical strength and guides BO via the EI acquisition function. A second GP model maps compositions to latent coordinates, linking compositions, latent coordinates, and yield stress. Together, these models enable the generation of candidate formulations along with their predicted yield-stress values.

An overview of our DR-GPR-BO pipeline is shown in Fig. 1. The workflow begins with preprocessing and standardization of all numerical variables, including composition variables, rheological observables, and yield stress, by centering each feature to zero mean and scaling to unit variance. This removes differences in physical units and relative scaling, thereby ensuring numerical stability for downstream processes. Dimensionality reduction is then applied to standardized rheological features to obtain low-dimensional latent coordinates representing the rheological state of each formulation. Two separate GP models were trained. The first GP was trained in the latent space to predict the mechanical strength of the dried sample, and BO guided by the expected improvement (EI) acquisition function was performed to identify promising optima in the reduced space. The second GP mapped composition variables to latent coordinates, enabling the identification of new formulations at optimized yield stress values.

### Rheological feature space structure and DR embedding assessment

Previous work has shown that the rheological feature set exhibits strong internal correlations, motivating dimensionality reduction for efficient latent modeling^11^. An exploratory examination of pairwise feature relationships (Supplementary Section S1 and Supplementary Figs. S1-S2) shows that many descriptors associated with moduli and temperature display strong linear correlations. However, several features, such as the minimum phase angle ($\delta _{\textrm{min}}$), the gelation temperature ($T_{\textrm{gel}}$), and the storage modulus at 60$^\circ$C ($G^{\prime \prime }_{60^{\circ }\textrm{C}}$), deviate from purely linear trends and demonstrate nonlinear relationships with the yield stress ($\sigma _y$). This coexistence of both linear and nonlinear relationships among rheological observables supports our strategy of employing t-SNE and UMAP, along with PCA serving as a linear baseline. Consistent with these observations, PCA captures over 90 % of the total variance using only two components, indicating that the dominant structure of the rheological feature space is well described by a low-dimensional linear subspace. For direct comparison across methods, the same two-dimensional latent space was therefore used for t-SNE and UMAP throughout this study. While higher-dimensional embeddings could be explored, two dimensions were found sufficient to capture the relevant structure in the present dataset.

PCA natively allows for the determination of the cumulative explained variance as a function of the number of the retained components, allowing for the selection of this number, but t-SNE and UMAP do not offer an equivalent variance-based criterion. Therefore, we assessed the structural fidelity of all reduced feature spaces and, to guide hyperparameter selection for the nonlinear methods, computed standard embedding-quality metrics, including the Silhouette score^45^, trustworthiness^46,47^, and a *k*-nearest-neighbor (k-NN) preservation metric based on neighborhood overlap. Themetrics summarized in Table 1 reveal that PCA achieves the highest overall structural fidelity for the present dataset, consistent with the strong linear correlations observed among many rheological observables. This result indicates that, within the experimentally sampled composition and processing domain, a substantial fraction of the variance is well described by a low-dimensional linear subspace, in line with previous observations from the same dataset^11^. As shown in Table 1, t-SNE exhibits strong local neighborhood preservation at small neighborhood sizes ($k=5$), consistent with its emphasis on local structure, while performance decreases at larger *k*. UMAP displays a more balanced behavior across neighborhood sizes ($k=5$ and $k=10$), preserving both local and intermediate-scale relationships. These results demonstrate that, although linear structure dominates the variance in this dataset, nonlinear embeddings can preserve meaningful rheological relationships.

**Table 1:** Embedding quality metrics for the two-dimensional latent spaces produced by PCA, t-SNE, and UMAP. Silhouette score, *k*-nearest-neighbor preservation, and trustworthiness quantify local and global structure preservation and are used to evaluate the suitability of each embedding for downstream regression and optimization.

Method	Silhouette score	*k*-NN preservation ($k=5$)	Trustworthiness ($k=5$)	Trustworthiness ($k=10$)
PCA	0.486	0.897	0.995	0.989
t-SNE	0.572	0.808	0.987	0.916
UMAP	0.515	0.656	0.953	0.914

However, because these unsupervised quality metrics do not actually quantify the performance of GPR in the generated latent spaces, hyperparameter selection cannot rely solely on them. We therefore adopted a two-stage screening procedure. First, hyperparameter settings that produced embeddings with poor neighborhood preservation or low trustworthiness were discarded. For the remaining candidate embeddings, we evaluated their suitability by training GP models on the resulting latent coordinates and comparing their predictive performance. This combined representation-prediction screening ensured that the selected latent spaces preserved meaningful rheological structure while also supporting accurate GP modeling and BO.

### Surrogate modeling

**Fig. 2:**
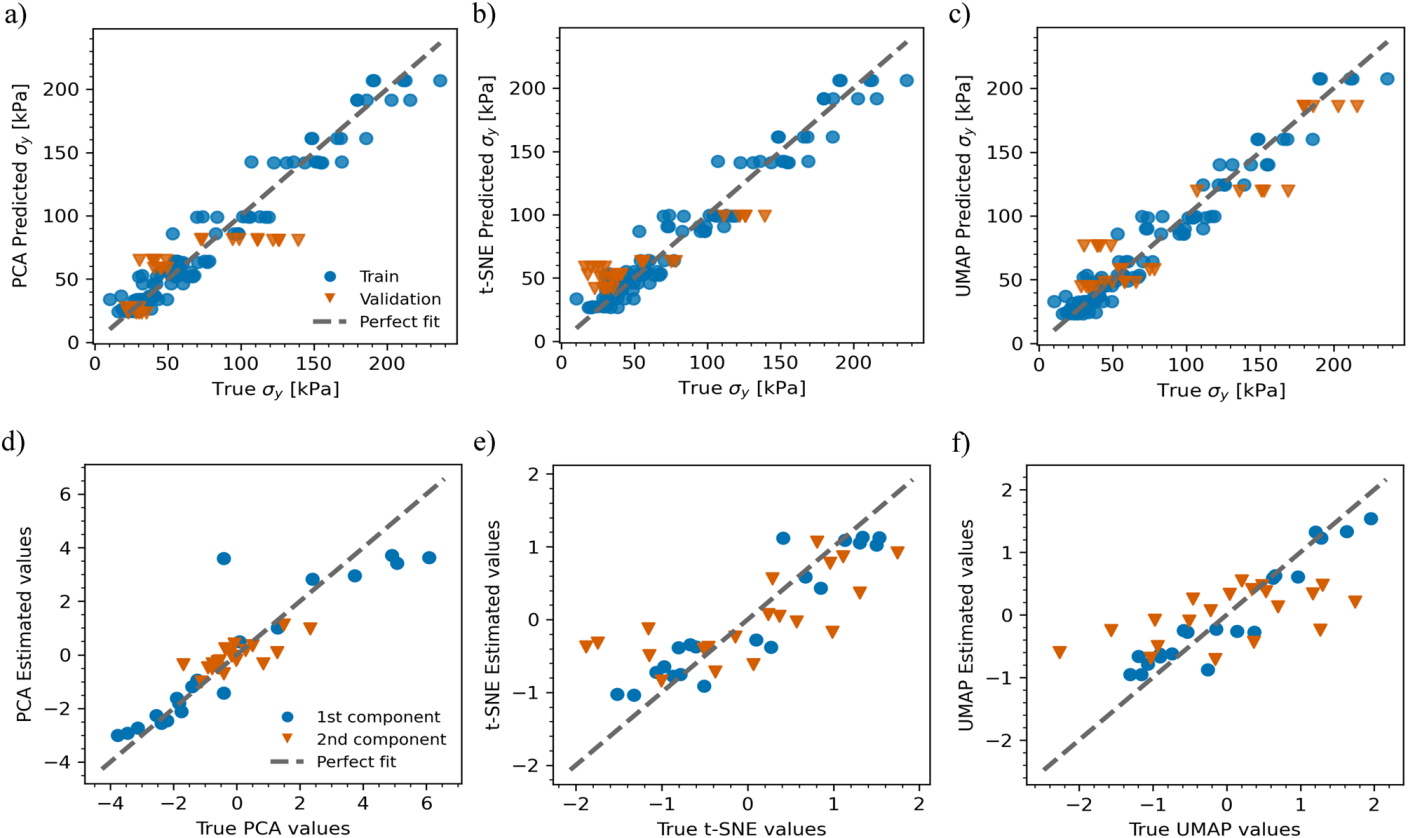
Performance of Gaussian process surrogate models. Panels a-c show the performance of the $\textrm{GP}_{\text {R2Y}}$ models predicting yield stress from latent rheological observables for (**a**) PCA, (**b**) t-SNE, and (**c**) UMAP. Predicted values are plotted against experimentally measured yield stress; circles denote training data and inverted triangles denote validation data. Panels d-f show the performance of the $\textrm{GP}_{\text {C2R}}$ models mapping composition variables to latent rheological observables for (**d**) PCA, (**e**) t-SNE, and (**f**) UMAP. Circles correspond to the first latent component and inverted triangles to the second latent component. Predicted latent components are plotted against their true values. For visualization only, t-SNE and UMAP latent coordinates were standardized to account for differences in component scaling; all model training and evaluation were performed on the original latent coordinates. Alignment along the diagonal indicates accurate reconstruction of the latent space.

Using the first GP model, denoted $\textrm{GP}_{\text {R2Y}}$, we trained surrogate models to predict the yield stress $\sigma _y$ of the dried foams from the latent coordinates produced by each DR method. To obtain an unbiased estimate of predictive performance, the dataset was split into training (80%) and validation (20%) subsets using a group-based strategy. This ensured that all replicate measurements of a given formulation appeared exclusively in either the training or validation set, preventing information leakage arising from repeated measurements of identical compositions. Across all DR methods, the surrogate models accurately predict experimentally measured yield stress values for both training and validation data (Figure 2a-c). The close alignment of predicted and true values along the diagonal indicates that the models capture the underlying structure of the rheological-mechanical relationship. Validation errors are comparable to training errors, suggesting that the models do not exhibit significant overfitting. The largest deviations from the diagonal are consistent with intrinsic sample-to-sample variability in the experimental yield stress measurements. Quantitatively, $\textrm{GP}_{\text {R2Y}}$ achieved validation root-mean-squared-error (RMSE) values of $\approx 23$ kPa across all DR methods, comparable to the intrinsic experimental variability of the yield stress measurements (19-25 kPa). Prediction errors below this range cannot be meaningfully distinguished from experimental noise. Across DR methods, the corresponding validation coefficient of determination ($R^2$) values ranged from 0.60 (PCA and t-SNE) to 0.86 (UMAP), consistent with the RMSE values and indicating that the surrogate captures the dominant rheology-mechanics relationship within the limits of experimental noise.

Because nonlinear DR methods do not provide a direct inverse transformation from latent space to the original feature space, an auxiliary GP model, denoted $\textrm{GP}_{\text {C2R}}$, was trained to map composition variables (MC and fiber fraction) to the corresponding latent coordinates. This model was trained on the set of unique formulations, since the inverse mapping is defined at the formulation level and experimental replicates do not provide additional independent information. Its performance was evaluated using an 80-20% random split of the unique formulations. Panels d–f in Fig. 2 show predicted versus true latent coordinates for this auxiliary model. Comparable trends across latent components indicate that both dimensions are learned with reasonable fidelity, although modest differences in predictive accuracy are observed between components. The first latent component was predicted with high accuracy ($R^2 = 0.73$-0.96 depending on the DR method), while the second component exhibited lower predictability. This behavior is expected, as the second latent dimension captures lower-variance directions of the rheological manifold. This mapping is necessarily approximate due to the non-uniqueness of the inverse problem, although it is sufficiently accurate to recover experimentally realizable compositions corresponding to optimized latent points within the domain supported by the training data. These results demonstrate that both t-SNE and UMAP can be effectively applied to regression tasks, provided they are properly tuned. Together, the two GP models establish a parallel surrogate modeling framework that links composition, the latent space of rheology observables, and the mechanical properties of the dried foam. The consistent behavior across training and validation sets confirms the robustness of the proposed DR-GPR framework.

### Bayesian optimization of the mechanical strength

**Fig. 3:**
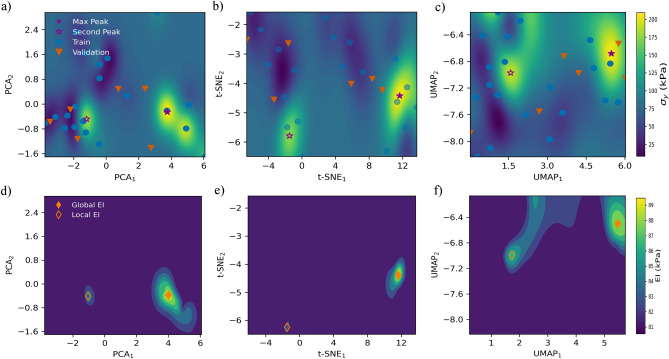
The top panels show the predicted yield stress $\sigma _y$ (colorbar) in 2D latent spaces for (**a**) PCA, (**b**) t-SNE, and (**c**) UMAP. Filled stars indicate the first peak and unfilled stars the secondary peak. Training and validation points correspond to the same split in Fig. 2. The lower panels show the EI (colorbar) derived from the plots in the top panels for (**d**) PCA, (**e**) t-SNE, and (**f**) UMAP. Filled diamonds indicate the primary peak and unfilled diamonds the secondary peak.

Contour maps of the predicted yield stress over each latent space (Fig. 3a–c) reveal a consistent two-peak landscape. The two regions of high predicted yield stress are separated by a valley of lower performance, suggesting the presence of two distinct formulation families. In line with our previous results^11^, this landscape appears across all DR methods, indicating that it reflects the underlying physical features rather than DR artifacts.

Bayesian optimization, here meaning the determination of the candidate optima based on the surrogate model and the uncertainty estimates, can be performed using the expected improvement acquisition function. In this study, EI was evaluated on a fixed latent-space grid as a diagnostic optimization criterion rather than as part of a sequential exploration strategy. We note that the maximum of the expected improvement surface coincides closely with a composition already present in the training dataset, reflecting the dense sampling of the composition space. As a result, the BO in this study primarily serves as a diagnostic tool for comparing DR methods and surrogate models, rather than identifying new compositions outside the sampled domain. Importantly, the predicted optima are comparable to, but do not exceed, the experimentally observed maximum yield stress, supporting the interpretation of the BO results as a consistency check rather than a discovery of improved formulations. The maxima of EI therefore indicate locations that balance high predicted yield stress with high model uncertainty, thereby providing a principled criterion for ranking candidate optima implied by the surrogate model. Posterior uncertainty was quantified directly from the $\textrm{GP}_{\text {R2Y}}$ predictive variance at these locations. The resulting EI contour maps (Fig. 3d–f) show two clear peaks around the maxima of the yield stress landscapes. The primary peak consistently exhibits both a higher predicted mean and substantially lower posterior uncertainty across all DR methods. In contrast, for PCA and t-SNE, although the secondary peak exhibits higher predictive uncertainty, its predicted mean is substantially lower than that of the primary peak. Because EI depends jointly on mean and uncertainty, the lower mean dominates, resulting in a smaller EI value at the secondary peak. In the UMAP embedding, the predictive uncertainties at the primary and secondary peaks are comparable; however, the higher predicted mean at the primary peak leads to a larger EI value. Consequently, the primary EI maximum represents the most robust trade-off between predicted performance and uncertainty. Accordingly, subsequent analysis and quantitative comparison focus on the primary EI optimum, which we take to correspond to the optimal composition.

### Compositional trends and physical interpretation

The latent-space coordinates corresponding to the EI maxima were mapped back to compositions using the $\textrm{GP}_{\text {C2R}}$ model. The resulting primary optimal compositions and predicted yield stresses are summarized in Table 2. These results demonstrate that for all DR methods, the primary EI maximum consistently corresponds to a compositional region characterized by relatively high MC and cellulose fiber contents ($\sim 1.4$ wt% MC and $\sim 1.9$ wt% fiber), yielding predicted yield stresses exceeding $203 \pm 35$ kPa on average. This region is associated with fiber-reinforced networks in which MC acts as a binder, enhancing mechanical integrity. A secondary, less prominent EI maximum is consistently associated with compositions featuring higher MC content and lower fiber contents ($\sim 1.8$ wt% MC and $\sim 0.4$ wt% fiber), with predicted yield stresses exceeding $174 \pm 43$ kPa. This region reflects MC-driven gelation mechanisms that promote mechanically robust closed-cell foam structures. Importantly, both compositional regimes align with experimentally observed trends and correspond closely to the distinct gelation and foam-formation regimes previously identified by Miranda et al.^11^. These results confirm that DR-assisted BO identifies physically meaningful formulation spaces, but the nonlinear methods do not show a clear improvement on the linear baseline.

**Table 2:** Comparison of optimized compositions and yield stress obtained from Bayesian optimization using different dimensionality reduction methods. Reported values correspond to the predictive mean ± one standard deviation of the GP posterior at the EI maximum. Experimental values are reported as mean ± standard deviation over repeated measurements.

Method	Composition (wt% MC, fiber)	Yield stress (kPa)
PCA	1.44, 1.96	$194 \pm 36$
t-SNE	1.36, 1.71	$209 \pm 19$
UMAP	1.49, 1.88	$206 \pm 51$
Experimental	1.50, 2.0	$193 \pm 16$

**Fig. 4:**
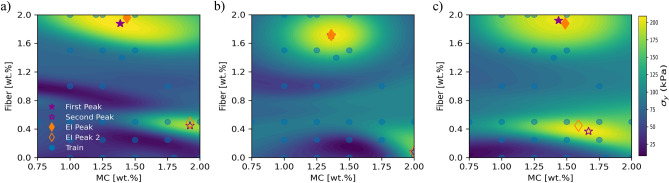
Composition-space contour maps of the predicted yield stress obtained using (**a**) PCA, (**b**) t-SNE, and (**c**) UMAP. Circles denote experimentally measured compositions used to train the $\textrm{GP}_{\text {C2R}}$ model. Star markers indicate the locations of the two yield stress maxima (exactly as in Fig. 3a–c), and the diamond markers indicate the EI optima (corresponding to Fig. 3d–f). The clustering of these points within two high-performance compositional regions highlights the physical relevance of the learned rheological manifolds.

In addition to the optimal point, one can also utilize the mapping between the composition and the latent space coordinates to plot the yield stress predictions over the full composition space considered (Fig. 4a-c). In these plots, both the latent-space yield stress maxima and the EI-selected optima are indicated. Although these composition-space contour maps were not used for iterative optimization, they provide a consistent geometric interpretation of the numerically identified optima. Notably, the four marked points (two yield stress maxima and two EI maxima) cluster within the same two high-performance compositional regions, indicating that the surrogate model does not expect improvement over the currently achieved optima.

An important question arising from the compositional analysis above is whether the learned rheological latent space remains predictive when the formulation chemistry is modified beyond the original training set, i.e. if the rheology-mechanical relationships captured by the DR-GPR-BO framework are transferable. To assess this, we considered an extended dataset comprising two additional experiments: one using a different fiber type in the suspension and another incorporating lignin into the suspension as an additive. These formulations introduce chemical and microstructural variations not present in the original dataset, while remaining within comparable rheological regimes. For each DR method, the rheological observables of these new formulations were first embedded into the corresponding latent space, after which the trained surrogate models were used to predict their yield stress values. In both cases, the predicted yield stress values obtained from all DR-based surrogates were in good agreement with the values reported by Miranda et al.^11^, with predicted yield stresses on the order of $\sim 100$ kPa for the lignin-containing foam (Supplementary Section S2). This agreement indicates that the auxiliary rheology-based latent space provides a robust basis for predicting mechanical performance even when compositional modifications are introduced, provided that the resulting rheologies lie within the manifold supported by the training data. These results demonstrate that the DR-GPR-BO framework is transferable to extended compositional domains, providing rheologists with a practical and data-efficient tool for exploring new biobased foam systems.

## Conclusions

This work evaluated the extent to which nonlinear dimensionality reduction methods can reliably support surrogate modeling and Bayesian optimization in the design of foams. A Gaussian process surrogate was used to predict yield stress, while BO was applied in a one-shot, non-sequential manner as a diagnostic tool for ranking candidate regions. Across PCA, t-SNE, and UMAP, the latent spaces constructed from rheological features enabled accurate prediction and optimization of the yield stress of dried foams. All methods converged on the same experimentally validated high-yield-stress formulation of approximately 1.5 wt% MC and 2.0 wt% fiber, demonstrating that DR-assisted BO is robust to the choice of dimensionality reduction method when nonlinear embeddings are properly tuned. The two-peak optimization landscape observed across all methods further indicates that this structure reflects underlying experimental trends rather than artifacts of DR. The latent structures identified by all DR methods correspond closely to known rheological and compositional regimes of methylcellulose-fiber systems^11^. One high-performance region is associated with simultaneously high MC and fiber contents, while the second corresponds to higher-MC, low-fiber formulations. The consistency of these regimes across DR methods suggests that the rheological features capture a strong, low-dimensional manifold that is preserved by nonlinear DR methods, although no significant improvement in predictive performance is observed for nonlinear methods in this dataset. The ability of the composition-to-latent surrogate to recover experimentally realizable formulations further supports the conclusion that rheology, rather than composition alone, provides a meaningful basis for predicting mechanical performance.

Among the nonlinear methods, UMAP preserved both local and global structures, while t-SNE provided strong local fidelity with slightly greater stochastic variability. Neither method provides a native inverse mapping, so the composition-to-latent surrogate introduces approximation error. However, in our case, the surrogate-based approximation was sufficiently accurate to recover valid formulations, indicating that the lack of strict invertibility does not preclude the use of nonlinear DR in BO pipelines for small experimental datasets.

Several limitations remain. The modest dataset size constrains the resolution of nonlinear manifolds and amplifies run-to-run variability in stochastic embeddings. The composition-to-latent mapping may not extrapolate reliably beyond the training domain, and DR methods can distort global geometry. As the optimization was performed on a fixed dataset, the identified optima lie within already sampled regions, and the framework does not assess the discovery of truly novel formulations. In a sequential, closed-loop setting, the choice of DR method could influence optimization through its effect on the model updates. Such effects were not investigated here and remain an important direction for future work. The comparable performance of PCA, t-SNE, and UMAP is influenced by the structure of the present dataset, which exhibits strong linear correlations among rheological observables and a limited number of formulations. In datasets with more complex nonlinear structures or higher variability, the differences between DR methods may become more pronounced.

Future work may also explore parametric DR approaches, neural networks with learned inverses, or physics-informed embeddings to address these challenges. Multiobjective optimization (e.g., balancing mechanical strength and fire resistance) and closed-loop experimental active learning platforms integrating automated rheology could further expand the utility of this framework. Overall, this study demonstrates that dimensionality-reduced rheological manifolds offer an effective and interpretable framework for data-efficient optimization of soft-matter formulations. By combining linear and nonlinear DR with GP modeling and BO, the framework presented here offers a practical route to accelerating the design of biobased foams and related rheology-governed materials.

## Methods

### Dataset and feature definitions

This study uses an experimental dataset of biobased methylcellulose (MC) and cellulose-fiber foams previously reported by Miranda et al.^11^, along with a complete description of the experimental methodology. It comprises 26 distinct MC-fiber formulations, with MC content ranging from 0.6 to 2.0 wt% and fiber content from 0.0 to 2.0 wt%. Each formulation was mechanically tested in five replicates to ensure robust characterization^11^. Each formulation is represented by a two-dimensional compositional vector $${\bf c} = \begin{bmatrix} c_{\textrm{MC}} \\ c_{\textrm{fiber}} \end{bmatrix} \in \mathscr {C}$$, where $c_{\textrm{MC}}$ and $c_{\textrm{fiber}}$ denote the weight fractions of methylcellulose and cellulose fiber in the liquid suspension, respectively. The rheological behavior of each suspension is described by an 11-dimensional feature vector ${\bf r} \in \mathscr {R}$ (observables explained in Supplementary Section S1), capturing viscoelasticity, gelation, and flow-related descriptors. The corresponding mechanical target variable is the scalar compressive yield stress $\sigma _y \in \mathscr {Y}$ of the dried foam.

### Dimensionality reduction and hyperparameter selection

PCA was used as a linear baseline and implemented following established procedures^11,34,35^. Unlike PCA, both t-SNE and UMAP require hyperparameter tuning^40,41^. Hyperparameters of nonlinear dimensionality reduction methods (e.g., t-SNE perplexity *PP*, UMAP number of neighbors $n_n$, and minimum distance $\delta$) were selected based on unsupervised embedding quality metrics and the downstream GP model’s performance. To compute embedding quality metrics, cluster labels are required for the Silhouette score only. For this purpose, we performed k-means clustering in each embedded space using a fixed number of clusters ($k=3$), chosen for diagnostic consistency across all embeddings rather than as a claim of intrinsic physical clustering. The Silhouette score was computed from these labels and is defined as $(b-a)/\max (a,b)$, where *a* is the mean distance to points in the same cluster and *b* is the mean distance to points in the nearest neighboring cluster. This metric evaluates how well samples are matched to their assigned clusters relative to alternative clusters^48^. A custom *k*-nearest-neighbor (*k*-NN) preservation metric was used to quantify the fraction of local neighborhoods in the original high-dimensional rheological feature space that are retained after dimensionality reduction. It is computed as the average Jaccard similarity between the sets of $k=5$ nearest neighbors in the original and embedded spaces. Trustworthiness measures the extent to which spurious neighbors are introduced during embedding; it penalizes points that appear close in the latent space but are distant in the original space, and was computed using the standard definition implemented in scikit-learn^49^. Together, these metrics provide complementary assessments of cluster coherence, local neighborhood preservation, and embedding distortion across PCA, t-SNE, and UMAP embeddings.

Two-dimensional embeddings were selected for all methods to enable direct comparison and to facilitate visualization and downstream surrogate modeling. Both t-SNE and UMAP are inherently stochastic and can produce slightly different embeddings across runs^41,48^. To minimize variability, we fixed random seeds, controlled initialization, and standardized threading across all runs. For both nonlinear DR methods, Euclidean distance was employed as the similarity metric, and PCA-based initialization was used to improve numerical stability and reproducibility of the embeddings. For UMAP, the number of nearest neighbors was set to $n_n = 13$, and the minimum inter-point distance was set to $\delta = 0.2$. For t-SNE, the perplexity was set to $PP = 9$ and Barnes-Hut approximation^50^ was used. These hyperparameter values were selected based on a combined evaluation of unsupervised embedding quality metrics and downstream GP regression performance, providing stable embeddings that balanced local neighborhood preservation with retention of global structure for the present dataset.

### Gaussian process modeling and Bayesian optimization

Two Gaussian process surrogate models were used in this work. The first model, $\textrm{GP}_{\textrm{R2Y}}$, maps the two-dimensional latent coordinates ${\bf z} \in \mathscr {Z}$, obtained from PCA, t-SNE, or UMAP, to the corresponding yield stress values $y \in \mathscr {Y}$. The second model, $\textrm{GP}_{\textrm{C2R}}$, provides an approximation mapping from composition ${\bf c} \in \mathscr {C}$ to latent coordinates ${\bf z}$, enabling the recovery of compositions corresponding to latent coordinates of interest. This model was trained at the level of unique formulations. To ensure consistency across dimensionality reduction methods, both GP models used the same kernel family, with hyperparameters optimized separately for each DR method. The covariance function was defined as

1 $$\begin{aligned} k({\bf x},{\bf x}') = k_{\textrm{c}} \, k_{\textrm{RBF}}({\bf x},{\bf x}') + k_{\textrm{w}} \delta ({\bf x}-{\bf x}'), \end{aligned}$$

where $k_{\textrm{c}}$ is a constant kernel controlling the signal variance, $k_{\textrm{RBF}}$ is a squared-exponential (radial basis function, RBF) kernel, and $k_{\textrm{w}}$ is the magnitude of a white-noise kernel implemented using the indicator function $\delta$ (which is one at zero, and zero otherwise). For $\textrm{GP}_{\textrm{R2Y}}$, an isotropic RBF kernel was used,

2 $$\begin{aligned} k_{\textrm{RBF}}({\bf z},{\bf z}') = \exp \!\left( -\frac{\Vert {\bf z}-{\bf z}'\Vert ^2}{2\ell ^2}\right) , \end{aligned}$$

reflecting the fact that the two latent coordinates lie in a common reduced space without independent physical meaning. For $\textrm{GP}_{\textrm{C2R}}$, an anisotropic RBF kernel with independent length scales $(\ell _{\textrm{MC}},\ell _{\textrm{fiber}})$ was used,

3 $$\begin{aligned} k_{\textrm{RBF}}({\bf c},{\bf c}') = \exp \!\left( - \sum _{m \in \{\textrm{MC},\,\textrm{fiber}\}} \frac{(c_m - c_m')^2}{2\ell _m^2} \right) , \end{aligned}$$

allowing the model to learn different sensitivities of the latent rheological coordinates to each compositional variable. In both GP models, the constant kernel amplitude, RBF length scale(s), and white-noise variance were treated as free hyperparameters and estimated by maximizing the log marginal likelihood (LML)^51^. The optimization was performed using the Limited-memory Broyden-Fletcher-Goldfarb-Shanno algorithm with bound constraints (L-BFGS-B)^52^. To mitigate convergence to local optima, multiple random restarts were employed (10 for $\textrm{GP}_{\textrm{R2Y}}$ and 100 for $\textrm{GP}_{\textrm{C2R}}$). For each restart, initial hyperparameters were sampled independently from uniform distributions defined over their respective bounds. For $\textrm{GP}_{\textrm{R2Y}}$, the constant kernel amplitude was constrained to the interval $[10^{-2},10^{2}]$, the RBF length scale to [0.5, 10], and the white-noise variance to $[5\times 10^{-2},1]$. For $\textrm{GP}_{\textrm{C2R}}$, the constant kernel amplitude was constrained to $[10^{-3},10^{3}]$, the anisotropic RBF length scales to $[10^{-2},10^{2}]$, and the white-noise variance to $[10^{-2},10]$. All bounds were defined a priori and used consistently during optimization to ensure numerical stability. The best solution across all restarts (highest LML) was selected. Each formulation was associated with five independent yield stress measurements sharing identical reduced embedding coordinates, reflecting repeated experimental measurements. Model performance (RMSE, MAE, and $R^2$) was computed by comparing the predictive mean of the GP against the individual experimental yield stress measurements on both training and held-out validation sets. Training metrics were used to assess fit, while validation metrics were used to evaluate generalization and to determine suitability for BO. Repeated measurements exhibited intrinsic yield stress variability of 19-25 kPa; prediction errors below this range cannot be meaningfully distinguished from experimental noise. Accordingly, surrogate models with validation RMSE values comparable to this scatter and $R^2 \gtrsim 0.6$ were considered sufficiently accurate for BO. The auxiliary model $\textrm{GP}_{\textrm{C2R}}$ was evaluated using the same metrics to assess the smoothness and internal consistency of the learned composition-latent mapping rather than predictive generalization in physical space. The optimized kernel hyperparameters for both GP models and all DR methods are summarized in Table 3.

**Table 3:** The values of optimized kernel hyperparameters for both the GP models across all DR methods. The RBF length scales are reported either as a single isotropic value for $\textrm{GP}_{\textrm{R2Y}}$ or as an anisotropic pair $(\ell _{\textrm{MC}}, \ell _{\textrm{fiber}})$ for $\textrm{GP}_{\textrm{C2R}}$. The white-noise variance $\sigma _{\textrm{n}}^2$ was treated as a free hyperparameter and optimized by marginal likelihood maximization in all cases.

Model	DR method	Length scale(s)	Noise $k_\textrm{w}$
$\textrm{GP}_{\textrm{R2Y}}$	PCA	$\ell = 0.50$	0.070
	t-SNE	$\ell = 0.99$	0.071
	UMAP	$\ell = 0.50$	0.060
$\textrm{GP}_{\textrm{C2R}}$	PCA	$(3.59,\,1.17)$	0.402
	t-SNE	$(1.85,\,1.31)$	0.415
	UMAP	$(2.93,\,1.56)$	0.88

Expected improvement was used as the acquisition function for evaluating candidate optima in the latent space. For a latent point ${\bf z}$ with the GP predictive mean $\mu ({\bf z})$ and standard deviation $\sigma ({\bf z})$, EI was computed in its standard form,

4 $$\begin{aligned} \textrm{EI}({\bf z}) = (\mu ({\bf z}) - \mu ^*)\,\Phi (Z) + \sigma ({\bf z})\,\phi (Z), \end{aligned}$$

where $Z = \frac{\mu ({\bf z}) - \mu ^*}{\sigma ({\bf z})}$, $\mu ^*$ is the best predicted mean of the $\textrm{GP}_{\textrm{R2Y}}$ model over the training inputs, and $\phi$ and $\Phi$ are the standard Gaussian probability density and cumulative distribution functions. EI was evaluated over a dense two-dimensional grid spanning each latent space to identify candidate optima. Posterior uncertainties were taken directly from the $\textrm{GP}_{\textrm{R2Y}}$ predictive variance, and the corresponding compositions were obtained using $\textrm{GP}_{\textrm{C2R}}$.

### Embedding of extended test data

To ensure consistency when embedding previously unseen test data (containing a different fiber type and a lignin additive), different strategies were adopted depending on the DR method. PCA and UMAP are parametric and therefore allow direct projection of new samples using the mappings learned during training. In contrast, t-SNE does not provide an explicit out-of-sample extension. To obtain a consistent latent representation in this case, the original dataset and the extended test dataset were embedded jointly using t-SNE, after which the corresponding low-dimensional coordinates were separated for subsequent analysis. This procedure ensured that all GP-based surrogate modeling and BO steps were performed within a single, coherent latent space, enabling an unbiased comparison across dimensionality reduction methods.

### Computational environment and reproducibility control

Beyond algorithmic stochasticity, nonlinear embedding methods can be sensitive to the surrounding computational environment, including library versions, numerical backends, and multi-threading behavior. During this study, minor changes in low-level dependencies were observed, which influenced the resulting embeddings, even when identical random seeds and hyperparameters were used. To ensure full reproducibility, we adopted a strict environment-control strategy. All random seeds were fixed at the Python, NumPy, and model levels; the number of computational threads was constrained; and all software dependencies were pinned to specific versions. After finalizing the pipeline, the entire computational environment was captured in a Docker container to eliminate system-level variability. All dimensionality reduction, regression, and Bayesian optimization results reported in this work were generated within this controlled environment. The corresponding Dockerfile and dependency specifications are provided with the accompanying code archive to enable exact reproduction of the reported embeddings and predictions. More details can be found in the Supplementary Section S3.

## Supplementary Information

Supplementary Information.

## Data Availability

All code and data used in this paper is available as Supplementary Information.
