# Supplementary material for: Nonlinear dimensionality reduction and Bayesian optimization for accelerating design of materials
Source: Sci Rep. 2026 May 9;16:21226. doi: 10.1038/s41598-026-51517-8 (PMC13346991; doi:10.1038/s41598-026-51517-8)
Supplement: Supplementary file 1 — Supplementary Information. [file 41598_2026_51517_MOESM1_ESM.zip › SI_folder_DR_v1/SI_DR_manuscript_V1.pdf]

Supplementary information

Nonlinear dimensionality reduction and Bayesian optimization for accelerating design of materials

Muhammad Osman Nadeem Farooqui[1,*], Isaac Y. Miranda-Valdez[1], Tero Mäkinen[1], Juha Koivisto[1], and Mikko J. Alava[1]

[1]Department of Applied Physics, Aalto University, P.O. Box 15600, 00076 Aalto, Espoo, Finland

[*]muhammad.farooqui@aalto.fi

February 18, 2026

This document is supplementary to the main manuscript "Nonlinear dimensionality reduction and Bayesian optimization for accelerating design of materials". It includes definitions of rheological features used in the study, extended exploratory visualizations, additional extended test set performance plots, and information on data and code availability.

S1 Rheological feature definitions

Ppreviously published biofoam data (see Ref. [1]), was used in this study. The rheology data consist of 11 rheological observables that characterize the viscoelastic response of methylcellulose-fiber mixtures on heating the system. These features include temperature variables, moduli, and crossover points. A complete list and detailed definitions are provided in Supplementary Table S1.

Exploratory feature relationships

To visually assess correlations and non-linear patterns among the 11 rheological features and their relevance to yield stress, we constructed pairwise scatter plots with kernel density estimates (KDEs) along the diagonals (Supplementary Figs. S1 and S2). This allowed both global and localized interactions to be observed. The following observations were made:

- Several modulus-related features, including $G'_{60^\circ\mathrm{C}}$, G'_{min}, and G''_{min}, showed high linear correlation, suggesting a degree of redundancy within the dataset, as expected.
- Temperature-based features such as $T_{G'_{\mathrm{min}}}$ and $T_{G''_{\mathrm{min}}}$ were strongly aligned, further supporting dimensionality reduction through linear projection.
- Conversely, δ_{min}, T_{gel}, and $G''_{60^\circ\mathrm{C}}$ exhibited nonlinear correlations, including their relationship with yield stress σ_y.
- Coloring the data by cellulose content (three quantile-based bins—low, medium, high—spanning the full 0–2 wt% composition range) revealed clustering behaviors, with high-cellulose formulations occupying narrow subspaces in T_{gel} and $G'_{60^\circ\mathrm{C}}$.

Symbol	Name	Description
$\delta_{\min}$	Minimum phase angle	Phase shift at the inflection point in $\delta(T)$; indicates the viscous–elastic balance during gelation onset.
$G'_{\delta_{\min}}$	Storage modulus at $\delta_{\min}$	Elasticity when $\delta(T)$ reaches its minimum, capturing matrix formation near gel point.
$G'_{25°C}$	Storage modulus at 25°C	Elasticity of the mixture at room temperature, relevant to foam processing.
$G'_{60°C}$	Storage modulus at 60°C	Elasticity near or after full gelation.
$G'_{\min}$	Minimum storage modulus	Lowest G' during heating, reflecting initial thermal softening of methylcellulose.
$G''_{25°C}$	Loss modulus at 25°C	Viscous component at room temperature.
$G''_{60°C}$	Loss modulus at 60°C	Viscous component of the gel phase at high temperature.
$G''_{\min}$	Minimum loss modulus	Lowest G'' during heating, also related to thermal softening.
$T_{G'_{\min}}$	Softening temp. (G')	Temperature at which G' reaches its minimum.
$T_{G''_{\min}}$	Softening temp. (G'')	Temperature at which G'' reaches its minimum.
T_{gel}	Gelation temperature	Temperature at which the material transitions into a gel; highly composition-dependent.

Supplementary Table S1. Descriptions of rheological observables used, adapted from Miranda-Valdez et al. [1].

These observations motivate the use of nonlinear DR approaches to comprehensively model the rheological landscape and its connection to mechanical performance.

S2 Generalization test: GPR predictions on independent extended datasets

The generalizability of the proposed DR-GPR-BO framework was evaluated using an independent test dataset not included in the primary training set, already published in Ref. [1]. This dataset comprises two additional experiments: one incorporating a different fiber type and another including lignin as an additive. To evaluate predictive performance without retraining, the test data were embedded into the latent spaces learned from the original dataset, as follows:

- **PCA and UMAP**: Both methods allow out-of-sample projection into an existing latent space. Test samples were therefore embedded using the mappings learned from the training data.
- **t-SNE**: As t-SNE does not support native out-of-sample projection, the training and test datasets were concatenated and embedded jointly, to ensure a consistent latent representation. After this, the latent space coordinates were separated for analysis.

Supplementary Figure S3a–c shows the first GP predictions obtained for the independent test data. The predicted yield stress values for the extended formulations agree well with reported experimental values, indicating that the trained surrogate models retain predictive accuracy for unseen composition spaces. Supplementary Figure S3d–f presents the corresponding EI landscapes

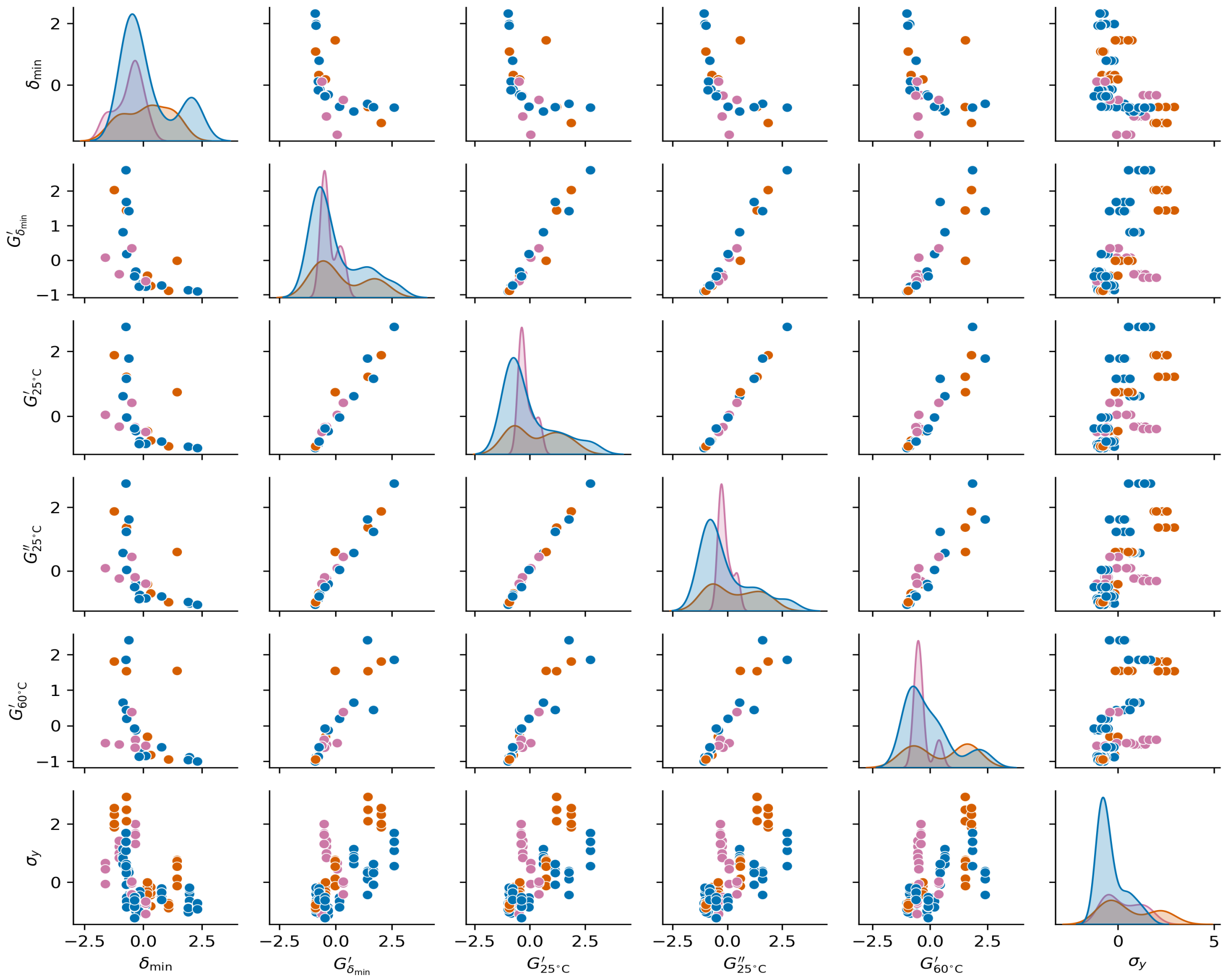

Supplementary Fig. S1. Pairwise scatter plots for the first five standardized rheological features, with KDEs along the diagonal. Data points are color-coded by methycellulose content level (blue for low, orange for medium, and pink for high).

incorporating the test data. Across all dimensionality reduction methods, the dominant EI peak remains consistent with that identified using the original dataset, confirming the robustness of the optimization outcome. For PCA and UMAP, both the predicted yield stress landscapes and EI maxima remain stable after inclusion of the test data. In contrast, minor shifts in secondary features are observed for t-SNE, reflecting the fact that its latent coordinates are recomputed when new samples are added. Importantly, the primary optimum remains unchanged across all methods, demonstrating that the conclusions of the BO analysis are not sensitive to the inclusion of additional formulations. These results indicate that the rheology-based latent space learned from the original dataset provides a robust basis for predicting mechanical performance under moderate compositional modifications, provided that the resulting rheological states lie within the manifold supported by the training data.

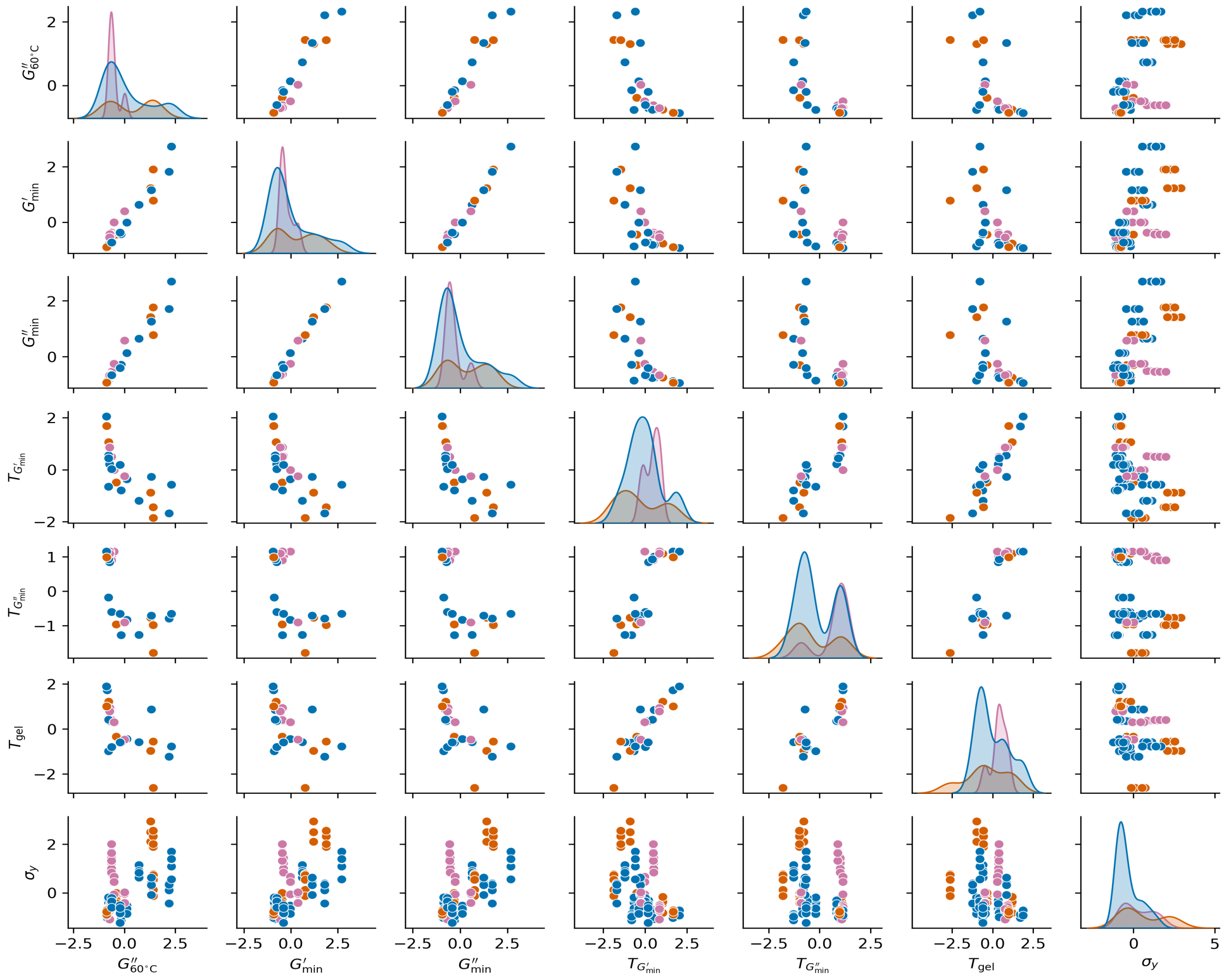

Supplementary Fig. S2. Same as Supplementary Figure S1 but for the rest of the standardized rheological observables, as well as the yield stress σ_y.

S3. Data and code availability

All data and source code required to reproduce the analyses presented in this work are provided as Supplementary Information. The supplementary zip archive contains: (i) the experimental dataset (`df_foams.csv`), (ii) three fully executable Jupyter notebooks implementing the PCA, t-SNE, and UMAP pipelines, namely

- `done_pca_supplementary_file_to_upload.ipynb`
- `done_tsne_supplementary_file_to_upload.ipynb`
- `done_umap_supplementary_file_to_upload.ipynb`,

and (iii) auxiliary files required to reproduce all figures, regression models, Bayesian optimization results, and inverse material design analyses reported in the manuscript. To run these notebooks successfully, place the dataset file `df_foams.csv` in the same directory as the notebooks.

All numerical experiments were executed in a controlled Python environment, as specified in the accompanying `requirements.txt` file. When executed with the provided settings and random seeds,

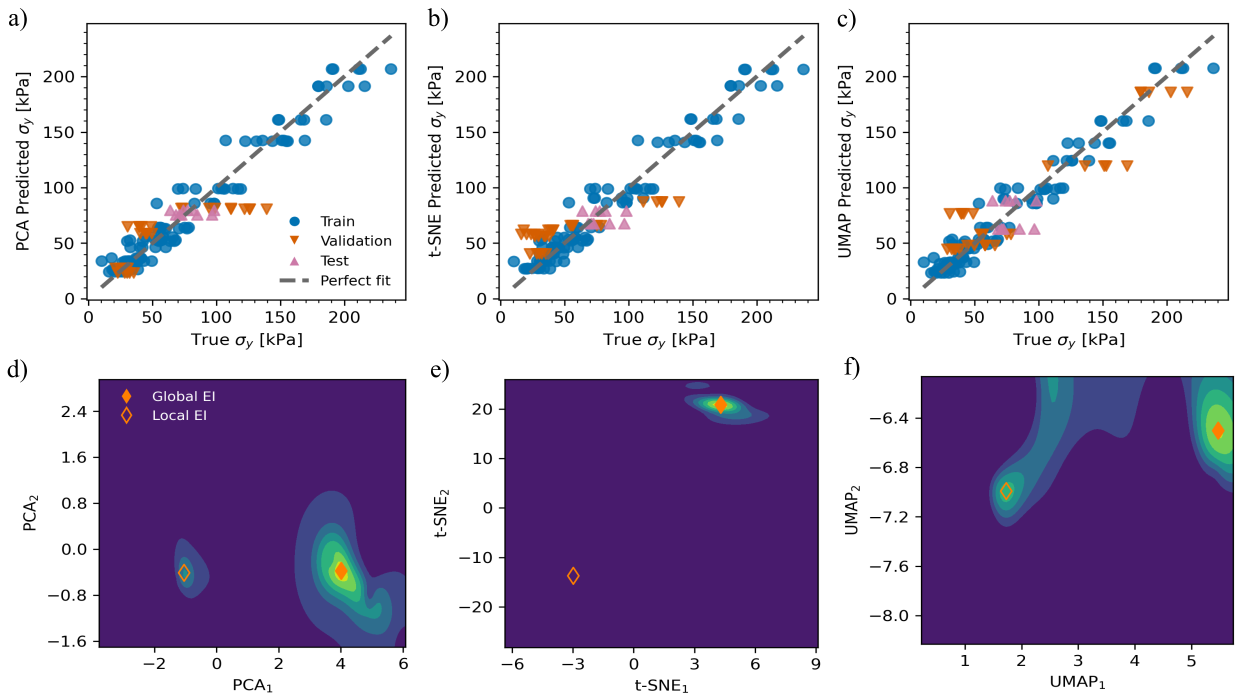

Supplementary Fig. S3. Top panels show the first GP predictions using additional test data. (a) Predicted vs. true yield stress values across all DR methods. Training and validation points follow the color scheme used in the main manuscript (Figure 2), while test points are highlighted as triangles. (b, c) GP-predicted yield stress values projected onto the t-SNE and UMAP latent spaces, respectively, using the same color coding as in panel (a). EI surfaces derived from the GP models using additional test data. (d) EI contour map in PCA space, identifying global and local optima. (b) EI surface in t-SNE space. (c) EI contour map in UMAP space. Color scales match those used in Figure 3 of the main manuscript. Consistent peak regions across all DR methods confirm the robustness of the optimization strategy.

the results are deterministic up to floating-point precision. All computations were performed using Python 3.12.2. The numerical environment was fully specified through pinned package versions provided in the supplementary `requirements.txt` file. For stochastic methods (t-SNE, UMAP, and GPR), fixed random seeds, deterministic initialization, and single-threaded execution were enforced to ensure reproducibility across platforms. To further guard against variability arising from future software updates or operating system differences, all scripts were additionally tested in a containerized Docker environment mirroring the computational setup used in this study. Identical qualitative and quantitative results were obtained inside and outside the container.

References

[1] Isaac Y Miranda-Valdez, Tero Mäkinen, Sebastian Coffeng, Axel Päivänsalo, Luisa Jannuzzi, Leevi Viitanen, Juha Koivisto, and Mikko J Alava. Accelerated design of solid bio-based foams for plastics substitutes. *Materials Horizons*, 2025. doi: 10.1039/d4mh01464b.
